# New Inventions

**Published:** 1912-09

**Authors:** 


### NEW INVENTIONS

Meredith Clark, Garden City, N. Y. 1,034,424. Measuring Device.

F. E. Stockelback, Detroit, Mich. Assignor to Frederick Stearns & Co., Detroit, Mich. 1,034,566. Process of Manufacturing Diacetyl-Paramido-Phenol.

A. A. Barratt, Claygate, Eng. 1,034,566. Nostril-Distending Apparatus.

A. W. Ekstrom, Los Angeles, Cal. 1,034,599. Process of Manufacturing Alkali Hydroxids.

G. H. Rabenalt, Dover, N. J. 1,034,646. Process of Purifying Hydrogen.

C. S. Ruckstuhl, St. Louis, Mo. 1,034,735. Thermometer.

Emil Hartwig, Jersey City, N. J. Assignor to Pomeroy Co., N. Y., N. Y. 1,034,793. Truss.

Chas. Ogilvy, New York, N. Y. 1,034,821. Mask used in Applying Anesthetics.

R. B. Higgins and Herman Albers, St. Louis, Mo. Assignors to Higgins, Albers Concrete Form Co., St. Louis, Mo. 1,034,913. Concrete Floor Decking.

M. W. Guioness, Philadelphia, Pa. 1,035,002. Bath-Cabinet.

W. J. Cameron, Enid, Okla. 1,034,417. Liquid-Fuel Burner.

Henry Hendricks, Alton, Ill. Assignor to F. R. Hendricks, Alton, Ill. 1,034,451. Rotary Gasolene Engine.

M. W. Johnson, Jr., Birmingham, Ala. 1,034,463. Gas Washer.

Chas. White, Baltimore, Md. 1,034,549. Gas Engine.

C. E. Brown, Columbus, Ohio. 1,034,577 Fuel-Oil Burner.

Max Glass, Vienna, Austria Hungary. 1,034,612. Lubricator.

F. E. Yates, Minneapolis, Minn. 1,034,669. Valve for Gas Burners.

B. M. Aslakson, Salem, Ohio. 1,034,673. Gas Engine.

W. C. Bosley, Houston, Tex. 1,034,682. Rotary Engine.

Reinhardt Daae, Pittsburgh, Pa. 1,034,695. Gas-Producer.

A. S. Freeman, Alexandria, Ind. Assignor to Freeman Mfg. Co., Alexandria, Ind. 1,034,702. Gas Generator.

Wm. Henry, Philadelphia, Pa. 1034,708. Valve for Internal Combustion-Engines.

J. W. Packard, Lakewood, N. Y. Assignor to Packard Motor Car Co., Detroit, Mich. 1,034,728. Water-Cooling System for Hydrocarbon-Engines.

J. H. Pierce, Bay City, Mich. Assignor to J. H. Budd, Wilmington, Del. 1,034,732. Engine.

L. T. Rhoades, Mont Clare, Pa. 1,034,835. Sparking Mechanism for Internal-Combustion Engines.

A. W. Bowden, Greenville, Me. 1,034,866. Safety Gas Burner.

David Brain, Los Angeles, Cal. 1,034,869. Steam Generator for Liquid Fuel Burners.

G. F. Clark, Daytona Beach, Fla. 1,034,874. Device for Removing Valve Springs.

H. E. Coffin and G. G. Behn, Detroit, Mich. Assignors to The Reynolds Motor Co., Detroit, Mich. 1,034,877. Rotary Valve for Explosion-Engines.

Eugene Deruntz, St. Louis, Mo. 1,034,889. Oil Burning Apparatus.

Jan H. Windemuller, Rotterdam, Netherlands. 1,035,127.

A. A. Low, Horseshoe and Harry Hertzberger, New York, N. Y. Said Hertzberg assignor to said Low. 1.035,614. Vaporizing Device for Explosive Engines.

A. C. Becker, Oakville, Can. Assignor to Waterbury Mfg. Co., Waterbury, Conn. 1,035,639. Inverted Burner.

F. H. Struble, New York, N. Y. Assignor to J. C. Klatzi, N. Y., N. Y. 1,035,653. Gasolene Filter.

H. C. Albrechy, Terre Haute, Ind., and J. P. McGee, Paris, Ill. 1,035,673. Vapor-Lamp.

J. E. Munyan, Deceased., Mantua, N. J. 1,035,807. Oil-Filter.

W. W. Dake, Muskegon, Mich. Assignor to Pyle-National Elec. Headlight Co., Chicago, Ill. 1,035,820. Elastic-Fluid Turbine.

F. G. Cottrell, Berkeley, and H. A. Burns, Oakland, Cal. Assignors to International Precipitating Co., San Francisco, Cal. 1,035,422. Apparatus for Separating Suspended Particles from Gaseous Bodies.

E. M. Lloyd, San Jose, Cal. Assignor to T. H. Ball, San Jose, Cal. 1,035,455. Burner Attachment.

J. H. T. Mills and V. H. Mills, Hubbard, Tex. 1,035,459. Oil-Burner.

E. A. Radunz and Otto Englehardt, Toledo, Ohio. 1,035,467. Safety-Valve for Gas-Lines.

J. N. Ruffin, New York, N. Y. 1,035,474. Caloric Engine.

B. F. Stewart, Chicago, Ill. 1,035,488. Gas Engine.

G. A. F. Ahlberg, Chicago, Ill. 1,035,513. Internal Combustion Engine.

T. M. Sandiford, Masterton, New Zeland. Assignor to The Automatic Gas Pressure Lamp Lighter Co., Ltd., Masterton, New Zealand. 1,035,477. Apparatus for Lighting and Extinguishing Gas Lamps.

Fred'k Dietz, New York, N. Y. Assignor to R. E. Deitz Co., New York, N. Y., and C. T. Ham Mfg. Co., Rochester, N. Y. 1,035,549. Tubular Lantern.

W. F. Goodwin and Geo. Dawson, San Francisco, Cal. 1,035,-576. Automatic Oil Stoker.

P. A. Guye and C. E. Guye, Geneva, Switzerland. 1,035,581. Apparatus for Producing Endothermic Reactions in Gases.

C. C. Keyser, Pensacola, Fla. 1,035,600. Internal Combustion Engine.

A. C. Stewart, Los Angeles, Cal. 1,035,651. Auxilary Air Valve for Carbureters.

H. F. Liedtke, Wilkinsburg, Pa. 1,035,713. Turbine Governor.

C. E. Arundel, Wash., D. C. Assignor to W. F. Robertsm, Wash., D. C. 1,035,137. Acetylene Gas Burner Tip.

W. W. Boa and Karl Schoenbauer, Grand Rapids, Mich. 1,035,150. Starting Device for Explosion Engines.

F. J. Madden, Albert Lea, Minn. Assignor to O. K. Earle, Minneapolis, Minn. 1,035,208. Automatic Cut-Off Valve for Gas Burners.

E. V. Reno and J. A. Bois, La Garenne-Colombes, France. Assignor to Sphinx Motor Co., New York, N. Y. 1,035,234. Internal Combustion Engines.

R. V. Smith, Salt Lake City, Utah. Assignor to Arvis Motor Co., Salt Lake City, Utah. 1,035,253. Rotary Engine.

John West, Southport, Eng. 1,035,287. Discharging and Charging Machine for Gas Retorts.

J. T. Bethel, So. Richmond, Va. 1,035,307. Lamp Burner.

D. E. Collard, Jefferson, Colo. 1,035,318. Ignition Apparatus for Explosive Engines.

P. W. Kane, Chicago, Ill. Assignor to Kane Machinery Co 1,035,356. Starting Device for Explosive Engines.

P. A. Painchaud, Plessisville, Que., Can. 1,035,383. Starting Mechanism for Internal Combustion Engines.

H. S. Simpson, Fairbury, Neb. Assignor to C. H. Shaffer, Fairbury, Neb. 1,035,391. Internal Combustion Engine Cylinder.

I. N. Lewis, U. S. Navy. 1,035,454. Internal Combustion Power Apparatus.

F. M. Ashley, New York, N. Y. 1,035,517. Gas Burner.

H. E. Curtis, West Hampstead, London, Eng. 1,035,163. Abdominal Support for Medical and Surgical Purposes.

O. H. McQuary, Jr., Lawrence, Kans. 1,035,217. Face Protector.

E. M. Smith, Martin, Mich. 1,035,252. Athletic Device.

Hermann Stumpf, Berlin, Ger. 1,035,261. Atomizer.

B. D. Knickerbocker, Chicago, Ill. 1,035,362. Bath Cabinet.

Richard Cartwright, Salem, Ore. 1,035,417. Truss.

Jan Steynis, Bay Shore, N. Y. Assignor to Steynis Ozone Co. 1,035,489. Apparatus for the Production of Ozone.

M. H. Turner, Washington, D. C. 1,035,500. Truss.

John Connery, St. Louis, Mo. 1,035,535. Medicated Air Compound.

J. J. DeGloria, Oakland, Cal. 1,035,546. Suppository.

Maria Rosse, Chicago, Ill. 1,035,642. Invalid Carrier.

S. E. Walton, Mountainair, N. Mex. 1,035,760. Crutch.

Edward Abadie, Leotard, Paris, France. 1,035,764. Hernial Bandage.

Victor Guinzburg, New York, N. Y. Assignor to I. B. Kleinert Rubber Co. 1,033,097. Baby Pants or Diaper-Cover.

Werner Siebert, Rheinfelden, Baden, Ger. 1,033,126, Method of Producing Enothermic Compounds from their Components.

John Barnes, Rockford, Ill. 1,033,147. Truss.

John Sklar, Brooklyn, N. Y. 1,033,627. Obstetrical Forceps.

J. A. Singmaster, Palmerton, Pa. 1,033,127. Manufacture of Producer-Gas.

O. P. Underwood and R. S. Hill, Des Moines, Iowa, said Underwood assignor to H. C. Mills, Des Moines, Ia. 1,033.130. Carbureter.

G. D. Dillon, Kansas City, Mo. 1,033,160. Explosive Engine.

E. F. Hall, Minneapolis, Minn. 1,033,314. Gas Cock Attachment.

Chas. Wiebke, Newark, N. J. 1,033,364. Antifluctuating System for Gas Distribution.

Adolfo De Clairmont, Toledo, O. 1,033,386. Attachment for Carbureters.

W. W. Kemp and W. H. Van Horn, Baltimore, Md. Assignors to C. M. Kemp Mfg. Co., Baltimore Md. 1,033,416. Gas Burner.

C. A. Morris and W. H. Merritt, Red Bank, N. J. 1,033,443. Carbureter.

M. C. White and O. C. Duryea, Los Angeles, Cal 1,033,503, 1,033,504. Internal Combustion Power Hammer.

J. D. Hopkins, Clinton, Ia. 1,033,588. Hot Water Heater.

F. J. Kaeser, Reno, Nev. 1,033,594. Oil Burning System.

J. B. Nau, New York, N. Y. 1,033,611. Gas Producer.

Han Wilhelm C. Schroder, J. H. Drager and Alex. B. Drager, Lubeck, Ger. Assignors to The Firm of Dragerwerk, Heinr & Bernh., Drager, Lubeck, Ger. 1,033,626. Apparatus for Testing Gas.

G. H. Waters, Greer, S. C. 1,033,641. Self Heating Sad Iron.

H. H. Hustead, Brown, W. Va. 1,033,700. Safety Gas Cut-Off.

Gabriel Iochum, Paris, France. Assignor to L. J. Guitard, Paris, France. 1,033,701. Rotary Explosion Engine.

E. K. Standish, Waltham, Mass. 1,033,748. Rotary Explosion Engine.

G. W. Hutchinson, Walton, New Zealand. 1,033,760. Internal Combustion Engine.

R. S. Dickinson, Los Angeles, Cal. 1,034,213. Rotary Internal Combustion Engine.

A. B. Cobb, Waterbury, Conn. 1,034,204. Purifier for Acetylene Gas.

H. L. Doherty, New York, N. Y. 1,034,215. Apparatus for Removing Suspended Matter from Gases and Vapors.

H. L. Doherty, New York, N. Y. 1,034,216. Apparatus for Removing Suspended Liquids and Solids from Gases.

C. G. Farez, Coney Island, N. Y. 1,034,224. Gas Burner.

J. T. Metcalfe and Garfield Metcalfe, Quincy, Pa. Assignors Minneapolis, Minn. 1,034,264. Gas Meter Leak Detector.

J. T. Metcolfe and Garfield Metcalfe, Quincy, Pa. Assignors to Quincy Engine Co., Quincy, Pa. 1,034,298. Gasolene Spray or Vaporizer Valve.

Wilber Proffitt and G. H. Brooks, Mars, Pa. 1,034,298. Automatic Gas Shut-Off Valve.

Robt. Runnion and Alonzo Runnion, Spencer, W. Va. 1,034,-304. Burner.

O. C. Dennis, Chicago, Ill. 1,033,788. Bust Form.

M. H. Kutch, Terre Haute, Ind. 1,033,808. Vaginal Douche Cup.

Nathan Sulzberger, New York, N. Y. 1,033,841. Derivatives of Metacresol with Organic Acids.

G. T. Fette, Cincinnati, Ohio. 1,033,883. Electromagnet for Surgical Purposes.

H. B. Schultz, Atlanta, Ga. 1,033,957. Abdominal Supporter.

Max Englemann, Elberfeld, Ger. Assignor to Farbenfabriken Vorm Friedr Bayer & Co., Elberfeld, Ger. 1,034,092. Pharmaceutical Products.

Max Englemann, Benedikt Merkel, and Georg Wesenberh, Elberfeld, Ger. Assignors to Harbenfabriken Vorm, Friedr, Bayer & Co., Elberfeld, Ger. 1,034,093. Disinfecting Agents.

A. C. Knowlson, Ann Arbor, Mich. 1,034,123. Nostril Expander.

Max Stern, Elberfeld, Ger. Assignor to Farbenfabriken Vorm Friedr, Bayer & Co., Elberfeld, Ger. 1,034,161. Cholesterin Preparation.

Ludwig Taub, Elberfeld, Ger. Assignor to Farbenfabriken, Vorm Friedr, Bayer & Co., Elberfeld, Ger. 1,034,166. Disinfecting Agent.

G. P. Vanier, Stellton, Pa. 1,034,170. Potash Bulb.

F. S. Young, Newark, N. J. 1,034,330. Process of Manufacturing Magnesium Carbonate.

W. J. Kappeler, deceased, Akron, O., by Mamie Kappeler, Executrix, Akron, Ohio. 1,034,388 Window Canopy.

H. H. Bunzel, Washington, D. C. 1,034,400. Method for Determining the Oxidase Content of Plant Juices.

Henry Collinet, Chicago, Ill. 1,033,783. Gasolene Engine.

J. B. Larson, Minneapolis, Minn. Assignor to E. T. Engstrom and W. M. Higley, Minneapolis, Minn., and J. B. Lee Company. 1,033,809. Tray for Gas Purifying Tanks.

C. W. Beck, Rockville Center, N. Y. Assignor to Oxweld Acetylene Co. 1,033,863, 1,033,864. Acetylene Gas Generator.

A. W. Hutchins, Providence, R. I. Assignor to W. C. Woodward, Providence, R. I. 1,033,897. Fuel Shipping Tank.

W. M. Gentle, Greenwood, Ind. 1,033,886. Carbureter.

W. S. Lee, Detroit, Mich. 1,033,911. Explosion Engine.

